# An explainable machine learning model for prognosis prediction in sudden sensorineural hearing loss under integrated therapy

**DOI:** 10.3389/fmed.2026.1845137

**Published:** 2026-06-01

**Authors:** Xiaoxiao Ye, Yuxin Deng, Binbin Xiong, Min Chen, Gang Chen, Chen Huang

**Affiliations:** 1 Faculty of Chinese Medicine, Macau University of Science and Technology, Taipa, Macao SAR, China; 2 Department of Otolaryngology-Head and Neck Surgery, Zhuhai Hospital of Integrated Traditional Chinese and Western Medicine, Zhuhai, China

**Keywords:** integrated therapy, machine learning, prognosis prediction, Shapley additive explanations, sudden sensorineural hearing loss, XGBoost

## Abstract

**Background:**

Sudden sensorineural hearing loss (SSNHL) is a common otologic emergency with variable prognosis. Reliable prognostic tools, particularly for patients undergoing integrated therapy, remain scarce.

**Methods:**

This retrospective study enrolled 227 patients with unilateral SSNHL of the Qi stagnation and blood stasis syndrome who received standardized integrated therapy. Patients were partitioned into training (70%) and validation (30%) sets via outcome-based stratified sampling. To prevent information leakage, all preprocessing and feature selection were conducted strictly within the training set. Eight machine learning models were optimized through five-fold cross-validation (primary metric: AUC). Model performance was systematically evaluated across discrimination, calibration, and clinical utility, with SHapley Additive ExPlanations (SHAP) analysis applied to quantify interpretability and identify key prognostic predictors.

**Results:**

The XGBoost model demonstrated superior performance in the validation set (AUC: 0.718; 95% CI: 0.590–0.846), with robust calibration and positive clinical net benefit. SHAP analysis identified four key predictors: activated partial thromboplastin time (APTT), disease duration, platelet count (PLT), and total protein (TP). Non-linear interpretability revealed distinct prognostic thresholds: a critical intervention window was identified within 10 days of symptom onset; optimal recovery was associated with PLT counts of 200–250 × 10^9^/L (risk increased >300 × 10^9^/L) and APTT values of 25–30 s (<25 s indicated higher failure risk). Furthermore, TP exhibited a tri-phasic association, with peak prognostic probability at 65–75 g/L, whereas hypoproteinemia (<60 g/L) and elevated levels (>75 g/L) correlated with reduced recovery likelihood.

**Conclusion:**

This study developed an explainable XGBoost-based model, providing an initial framework for prognostic prediction in SSNHL patients. It demonstrates the integration of machine learning with TCM-informed therapy, suggesting potential pathways for personalized care. While promising, the results should be interpreted with caution due to the lack of external validation. Further multicenter prospective studies are crucial to confirm the model’s generalizability and validate the identified biological predictors.

## 1 Introduction

Sudden sensorineural hearing loss (SSNHL) is a common emergency in otolaryngology, defined as sensorineural hearing loss of ≥20 dB HL occurring in at least 2 consecutive frequencies within 72 h without a clear identifiable cause (1). In the theoretical system of Traditional Chinese Medicine (TCM), this disease falls under the category of “Bao Long (Sudden Deafness)” (2), among which Qi stagnation and blood stasis syndrome is a common clinical syndrome type (3). Its core pathogenesis is highly consistent with the pathophysiological mechanism of “inner ear microcirculation disturbance” described in Western medicine (4–6), and integrated Chinese and Western medicine treatment based on this syndrome type has shown definite efficacy in clinical practice (7).

Hearing impairment affects over 1.5 billion people worldwide, and the incidence of SSNHL has increased in recent years (8). Even with standardized treatment, the prognosis of the disease remains highly variable, and a considerable proportion of patients cannot achieve complete hearing recovery (9, 10) This heterogeneity in prognosis indicates that there is an urgent clinical need for reliable tools to predict treatment response and provide guidance for early clinical decision-making.

Current prognostic models for SSNHL have several limitations. First, most models are constructed solely based on data from patients receiving conventional Western medicine treatment (11), with insufficient consideration of integrated Chinese and Western medicine treatment regimens. Second, individual heterogeneity among patients, especially differences in clinical classification, will reduce the accuracy and generalizability of the models (12). Third, a large number of machine learning models are “black-box models” with insufficient interpretability, making it difficult to implement them in clinical scenarios (13).

Integrated treatment regimens combining conventional Western medicine and traditional TCM therapies are now increasingly widely used in clinical practice (14), especially in East Asia. However, specific prognostic models tailored to this integrated treatment setting are extremely scarce. In addition, there are still very few relevant studies on explainable machine learning models that can simultaneously take into account high predictive performance and output clinically interpretable evidence.

Therefore, this study aims to construct and validate an explainable machine learning model to predict the prognostic outcomes of SSNHL patients receiving integrated therapy. The study integrates multiple machine learning algorithms, combined with SHapley Additive ExPlanations (SHAP)-based interpretability analysis, to explore key prognostic factors (15). Meanwhile, we aim to develop a clinically implementable tool to realize early risk stratification and the formulation of individualized treatment regimens.

## 2 Materials and methods

### 2.1 Study design and ethical approval

This was a single-center retrospective cohort study conducted at Zhuhai Hospital of Integrated Traditional Chinese and Western Medicine. Consecutive patients with a confirmed diagnosis of SSNHL admitted between January 2023 and August 2025 were enrolled. All patients completed at least 12 weeks of follow-up, with no loss to follow-up.

This study was conducted in strict accordance with the Declaration of Helsinki, and was approved by the Ethics Committee of Zhuhai Hospital of Integrated Traditional Chinese and Western Medicine (Approval No.: 2025–03-008-F01). Given the retrospective nature of the study and the use of de-identified data, a waiver of informed consent was granted by the ethics committee. This study adheres to the TRIPOD-AI reporting guidelines for predictive modeling.

### 2.2 Study subjects

#### 2.2.1 Diagnostic criteria

SSNHL was diagnosed in accordance with the Guidelines for the Diagnosis and Treatment of Sudden Deafness (2015) (1), defined as a sudden hearing loss of ≥20 dB HL affecting at least 2 consecutive frequencies occurring within 72 h, with no clear identifiable pathogenic cause.

Combined with traditional diagnostic criteria, patients were further classified into the specific clinical subtype of Qi stagnation and blood stasis syndrome in strict accordance with the syndrome differentiation criteria from *Chinese Otorhinolaryngology*, based on symptoms including sudden hearing loss and tinnitus, as well as TCM signs related to tongue manifestation and pulse condition (2). The diagnostic results were independently reviewed and confirmed by two senior attending TCM physicians, with discrepancies resolved by a third chief TCM physician, with high inter-rater reliability (Cohen’s kappa coefficient *κ* = 0.89, indicating almost perfect inter-rater agreement per Landis-Koch criteria), indicating excellent consistency of the diagnostic results. The diagnostic process followed authoritative national standardized TCM syndrome differentiation criteria to ensure reproducibility.

Patients were eligible for inclusion if they met all of the following criteria:

(1) Met both the above Western medicine diagnostic criteria for SSNHL and the TCM syndrome differentiation criteria for Bao Long (Sudden Deafness) with Qi stagnation and blood stasis syndrome;
(2) Aged between 18 and 75 years old;
(3) Otoscopy showed unobstructed external auditory canal and intact tympanic membrane;
(4) Had complete pure-tone audiometry results at admission baseline and 12 weeks after disease onset during follow-up;
(5) Had complete clinical data and completed standardized follow-up.

Patients were excluded if they presented with any of the following conditions:

(1) Had a clearly identifiable cause of hearing loss (e.g., Meniere’s disease, tumors);
(2) Had a prior history of hearing impairment;
(3) Had bilateral SSNHL;
(4) Had severe systemic diseases that may affect laboratory test indicators.
(5) Missing key data.

**Figure 1:**
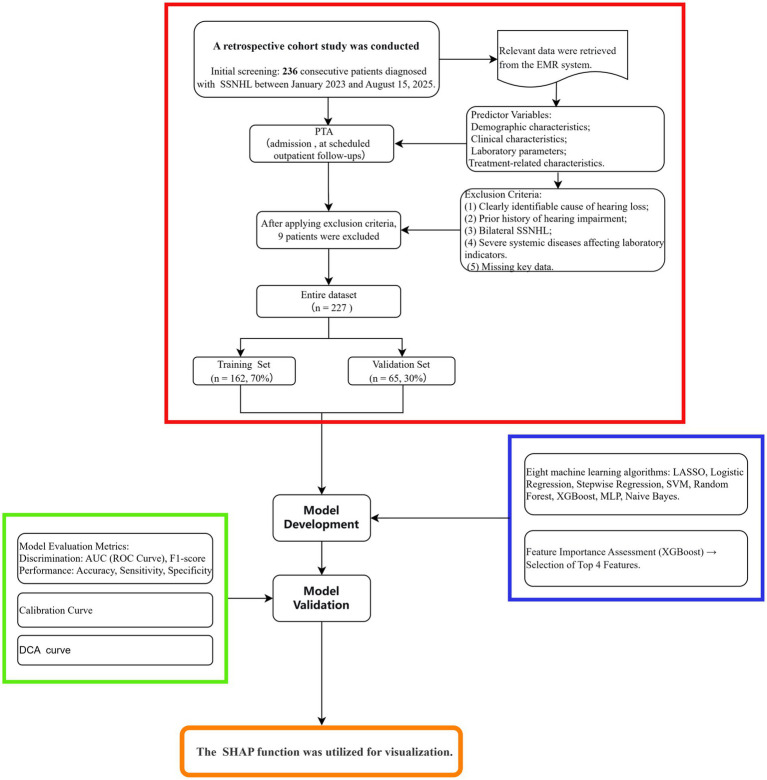
Flowchart of patient selection and study design. SSNHL, sudden sensorineural hearing loss; EMR, electronic medical record; PTA, pure tone audiometry; MRI, magnetic resonance imaging.

The detailed patient selection process is illustrated in Figure 1.

### 2.3 Treatment regimens

All patients received standardized integrated therapy, including conventional pharmacological treatment (e.g., systemic glucocorticoids, microcirculation-improving agents, and neurotrophic drugs), along with adjunctive interventions based on individualized clinical assessment.

Treatment strategies were determined at admission and remained unchanged throughout the study period, ensuring that all treatment-related variables were defined prior to outcome occurrence and minimizing potential information leakage.

### 2.4 Outcome measures

The final efficacy evaluation was performed at 12 weeks after disease onset in this study. The core efficacy endpoint was based on the difference in pure-tone average (PTA) of impaired frequencies between the baseline pure-tone audiometry results before admission treatment and the final follow-up pure-tone audiometry results at 12 weeks after disease onset. Efficacy was classified into 4 grades in accordance with the Guidelines for the Diagnosis and Treatment of Sudden Deafness (2015):

(1) Recovery: Hearing of impaired frequencies returned to normal level, the level of the healthy ear, or the baseline level before disease onset.
(2) Marked improvement: Average hearing of impaired frequencies improved by ≥30 dB HL compared with baseline.
(3) Improvement: Average hearing of impaired frequencies improved by 15 to <30 dB HL compared with baseline.
(4) No improvement: Average hearing of impaired frequencies improved by <15 dB HL compared with baseline.

In this study, patients with recovery, marked improvement, and improvement were combined into the treatment response group, and patients with no improvement were defined as the treatment failure group. This was used as the binary outcome variable for model prediction.

### 2.5 Predictor variables

All predictor variables were baseline data collected at admission prior to outcome occurrence. Treatment-related variables were predefined at admission and recorded before treatment initiation to avoid information leakage. Data were extracted from the Hospital Information System (HIS), Laboratory Information System (LIS), imaging system, and audiometry records. A total of 53 candidate variables were included and categorized into four groups:

(1) Demographic characteristics: sex and age.
(2) Clinical characteristics: affected ear side, disease duration (defined as the interval from symptom onset to admission), length of hospital stay, accompanying symptoms (tinnitus, vertigo, aural fullness), comorbidities (hypertension, diabetes mellitus), and cranial MRI findings.
(3) Laboratory parameters: including hematological indices (e.g., white blood cell count, platelet count, mean platelet volume), coagulation markers (e.g., prothrombin time, activated partial thromboplastin time, fibrinogen), biochemical indicators (e.g., electrolytes, blood glucose, blood urea nitrogen, serum creatinine), and liver function parameters (e.g., total protein, albumin, transaminases).
(4) Treatment-related characteristics: including standardized pharmacotherapy (e.g., systemic glucocorticoids, microcirculation-improving agents, neurotrophic drugs) and adjunctive interventions (e.g., local steroid administration, tinnitus therapies, vestibular rehabilitation, and TCM-based therapies such as herbal decoctions and external treatments).

#### 2.5.1 *A priori* variable exclusion criteria

To preserve methodological rigor, avoid outcome leakage, and strictly align with the study’s pre-specified core scientific aims, two categories of variables were excluded prior to model development:

(1) Outcome-related variables: Follow-up audiometric data (e.g., 12-week pure-tone average, PTA) were excluded to prevent label leakage, as these data directly constituted the basis for our primary efficacy endpoint definition.
(2) Baseline audiometric predictors: Although baseline PTA, hearing loss severity, and hearing loss type are well-recognized independent prognostic factors for SSNHL (with complete and accessible records in our original clinical dataset), they were intentionally excluded *a priori*. This study specifically aimed to evaluate the independent auxiliary prognostic value of non-audiometric routine indicators, which can be obtained immediately upon hospital admission without specialized otolaryngology audiometric examinations. Excluding these dominant predictive factors allows for an unbiased, clear assessment of the independent prognostic contribution of routine laboratory and clinical parameters, which is the core objective of this investigation.

All preprocessing steps, including feature selection and normalization, were performed strictly within the training set to further prevent information leakage.

### 2.6 Data preprocessing and dataset partitioning

The dataset was randomly partitioned into a training set (70%) and a validation set (30%) using stratified sampling based on the outcome variable. Baseline comparability between the two sets was confirmed by univariate analysis (all two-tailed *p* > 0.05). To ensure rigorous model evaluation, the validation set was strictly held out and excluded from all feature selection and model training processes.

#### 2.6.1 Missing data handling

Missing data were first quantified at both the patient and variable levels. Nine patients had at least one missing value, accounting for 3.81% of the cohort. At the variable level, 16 variables had missing values, with variable-level missingness ranging from 0.85 to 2.54%; no variable had more than 5% missingness. Therefore, complete-case analysis was used for the primary analysis.

To assess whether missing-data handling materially affected the data structure, multiple imputation by chained equations (MICE) was additionally performed as a sensitivity analysis. The distributions of key continuous variables and the proportions of major categorical variables were compared before and after imputation, which confirmed that MICE imputation did not materially alter the original data distribution (Supplementary Table S2).

All preprocessing steps, including feature selection and normalization, were conducted exclusively within the training set to prevent information leakage.

### 2.7 Model construction

Eight types of machine learning models corresponding to different algorithmic strategies were constructed in this study, specifically including: linear interpretable models (Logistic Regression, Least Absolute Shrinkage and Selection Operator (LASSO) Regression, Stepwise Regression), non-linear machine learning models (Support Vector Machine (SVM), Random Forest, Extreme Gradient Boosting (XGBoost), Multi-Layer Perceptron (MLP)), and rapid screening model (Naive Bayes).

Model tuning was performed within the training set using 5-fold cross-validation, and AUC was used as the primary optimization metric. For the RF model, the caret implementation was used and the number of trees was set to 800. The RF model was trained using all candidate predictors because RF performs embedded variable selection through random feature sampling at each split. However, no explicit pruning or tree-complexity restriction parameters, such as maxnodes, nodesize, or max.depth, were specified in the original RF model.

For XGBoost, the caret xgbTree implementation was used, and model selection was based on cross-validated AUC within the training set. The final model was selected according to validation-set performance, model stability, and parsimony.

Given the balanced outcome distribution (52.0% vs. 48.0%), no class weighting or resampling techniques were required during model training.

### 2.8 Feature selection strategy

Feature selection was performed separately within each pre-specified modeling framework using only the training set, in order to avoid information leakage. For each model, candidate predictors were selected or ranked according to the feature-selection procedure intrinsic to that modeling approach. The resulting model-specific feature subsets were then used to train the corresponding models. Model performance was evaluated on the validation set, and the final model was selected by considering predictive performance, model parsimony, clinical interpretability, and the events-per-variable (EPV) principle.

To evaluate the stability of the selected predictors under sample fluctuation, a bootstrap-based variable stability analysis was additionally performed using all candidate predictors. In each bootstrap-resampled training dataset, the pre-specified model framework was refitted, and the importance ranking of each predictor was recorded. We calculated the top-5 and top-10 selection frequencies of each predictor to assess stability.

Because some predictors showed relatively lower stability in the bootstrap analysis, an additional sensitivity analysis was performed by comparing the predictive performance of the original final optimal model with a reduced model excluding the low-stability predictor(s).

### 2.9 Model evaluation

Performance metrics were calculated in both the training set and the validation set. Training-set metrics were interpreted as apparent performance because they were calculated by applying the fitted models back to the training data. Therefore, validation-set performance was considered the primary criterion for model comparison and final model selection. Model performance was evaluated from three dimensions:

- Discrimination: assessed using the Area Under the Receiver Operating Characteristic Curve (AUC), accuracy, sensitivity, specificity, and F1-score.
- Calibration: evaluated using calibration curve, Brier score, and Hosmer–Lemeshow goodness-of-fit test.
- Clinical utility: assessed using Decision Curve Analysis (DCA).

### 2.10 Model interpretability

The SHAP method was used to assess model interpretability in this study, which can quantify the contribution of each feature to the model prediction results. Global feature importance, marginal effects, and interaction effects were analyzed to improve the clinical interpretability of the model and screen out key prognostic factors.

### 2.11 Statistical analysis and software tools

Continuous variables were expressed as mean ± standard deviation (SD) or median (interquartile range, IQR) as appropriate. Group comparisons were performed using the independent samples t-test or Mann–Whitney U test. Categorical variables were presented as counts (percentages) and compared using the chi-square test or Fisher’s exact test.

A two-sided *p* < 0.05 was considered statistically significant.

All analyses were conducted using R software (version 4.4.0). Key packages included caret, XGboost, iml, ggplot2, and stats.

## 3 Results

### 3.1 Baseline characteristics of the study population

A total of 236 patients were initially screened, and 227 patients were finally included in the analysis after excluding those with missing data. The study cohort comprised 104 males (45.8%) and 123 females (54.2%), with a median age of 42.0 years (interquartile range, IQR: 32.0–54.5).

Patients were randomly divided into a training set (*n* = 162) and a validation set (*n* = 65) using stratified sampling. No statistically significant differences were observed in all baseline characteristics between the two groups (all *p* > 0.05), indicating good comparability between the two sets. Detailed baseline data are presented in Supplementary Table S1.

### 3.2 Comparison of baseline characteristics between the treatment response and failure groups

Based on the efficacy evaluation at 12 weeks after disease onset, 227 patients were divided into the treatment response group (*n* = 118, 51.98%) and the treatment failure group (*n* = 109, 48.02%).

Significant differences were observed in demographic and clinical baseline characteristics. Specifically, patients in the response group were significantly younger (39.00 [30.00–50.00] vs. 49.00 [36.00–58.00] years, *p <* 0.001) and presented with a shorter disease duration (3.00 [2.00–6.75] vs. 5.00 [3.00–8.00] days, *p* < 0.001) compared to the failure group. No significant differences were found regarding sex distribution, affected side, or associated symptoms, including vertigo and dizziness (all *p* > 0.05).

Regarding treatment modalities, the response group had a higher rate of tinnitus-specific medication use (45.8% vs. 30.3%, *p* = 0.020). Conversely, the failure group showed higher utilization of psychotropic medications (51.4% vs. 36.4%, *p* = 0.032), Chinese patent medicines (55.0% vs. 41.5%, *p* = 0.047), and thrombolytic therapy (57.8% vs. 39.8%, *p* = 0.008). Notably, these treatment-related discrepancies likely reflect clinician decisions based on initial disease severity or symptom complexity rather than direct causal effects on therapeutic outcomes.

**Figure 2:**
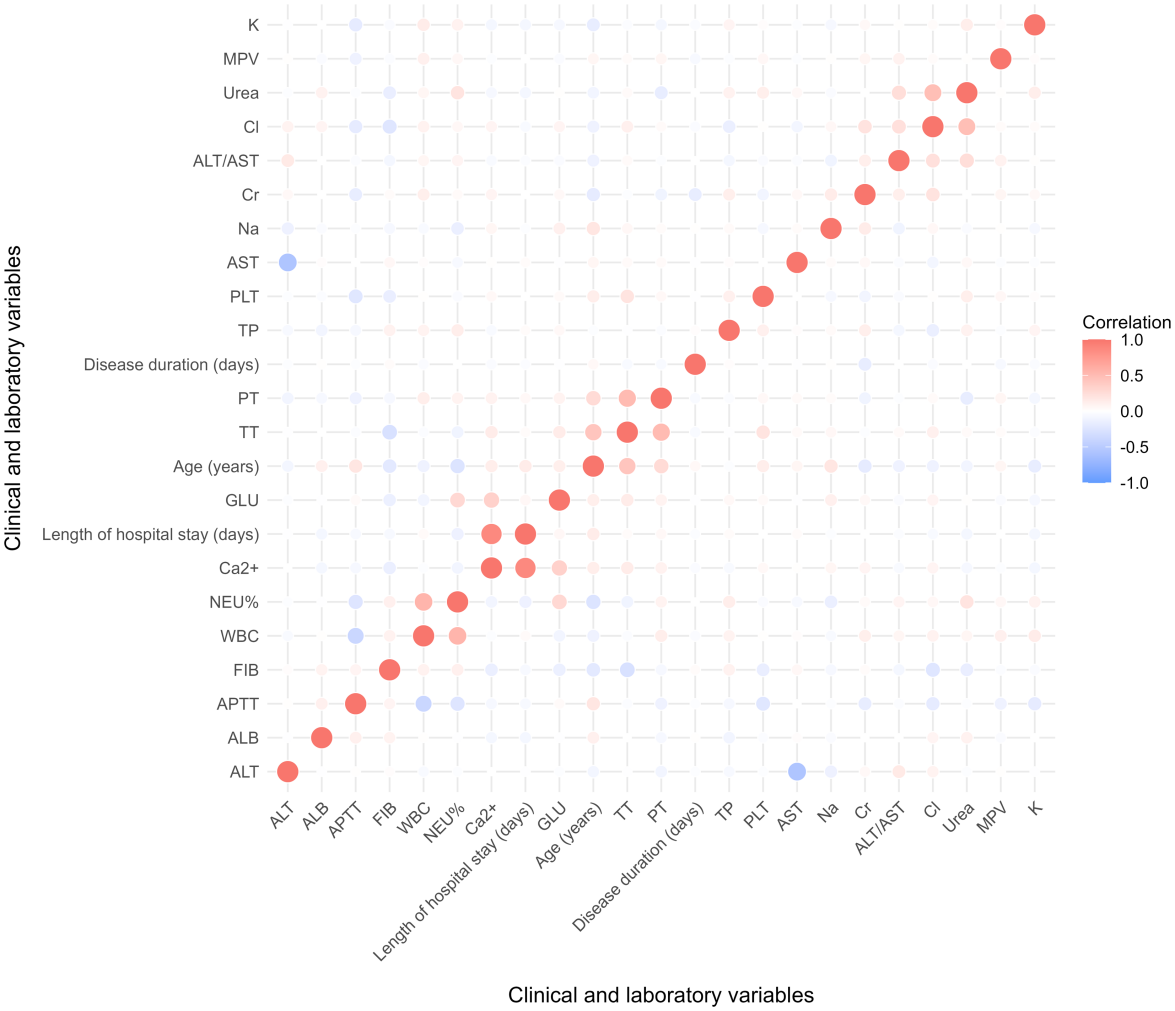
Correlation heatmap of continuous independent variables. The color gradient represents the strength of correlation, with 1.0 indicating a perfect positive correlation and −1.0 indicating a perfect negative correlation. WBC, White Blood Cell (Count); NEUT, Neutrophil (Percentage/Count); PLT, Platelet (Count); MPV, Mean Platelet Volume; PT, Prothrombin Time; APTT, Activated Partial Thromboplastin Time; TT, Thrombin Time; FIB, Fibrinogen; K, Potassium; Na, Sodium; Cl, Chloride; Ca, Calcium; GLU, Glucose; BUN, Blood Urea Nitrogen; Cr, Creatinine; TP, Total Protein; ALB, Albumin; ALT, Alanine Aminotransferase; AST, Aspartate Aminotransferase; ALT/AST, Alanine Aminotransferase/Aspartate Aminotransferase Ratio.

In terms of laboratory parameters, the response group exhibited significantly higher levels of mean platelet volume MPV (9.55 [9.10–10.40] vs. 9.30 [8.80–10.10] fL, *p = 0.033*) and activated partial thromboplastin time (APTT)(27.70 [26.22–29.90] vs. 26.90 [24.60–29.50] s, *p =* 0.035), alongside lower blood urea nitrogen (BUN) levels (4.81 [4.06–5.50] vs. 5.26 [4.34–6.17] mmol/L, *p* = 0.013). No other laboratory parameters reached statistical significance (all *p* > 0.05). Detailed results are presented in Table 1 and Figure 2.

**Table 1:** Comparison of clinical characteristics between the treatment-effective and treatment-ineffective groups of SSNHL patients.

Characteristics	Total (*n* = 227)	Treatment failure (*n* = 109)	Treatment response (*n* = 118)	*p-*value
Patient characteristics				
Sex [*n* (%)]				0.351
Male	104 (45.8)	46 (42.2)	58 (49.2)	
Female	123 (54.2)	63 (57.8)	60 (50.8)	
Age (median[IQR]), years	42.00 [32.00, 54.50]	49.00 [36.00, 58.00]	39.00 [30.00, 50.00]	<0.001
DD (median[IQR]), days	4.00 [2.00, 7.00]	5.00 [3.00, 8.00]	3.00 [2.00, 6.75]	<0.001
LOS (median[IQR]), days	7.00 [6.00, 9.00]	7.00 [6.00, 10.00]	7.00 [6.00, 9.00]	0.112
Affected side				0.691
Left	112 (49.3)	52 (47.7)	60 (50.8)	
Right	115 (50.7)	57 (52.3)	58 (49.2)	
Associated symptom				
Tinnitus				0.864
No	41 (18.1)	19 (17.4)	22 (18.6)	
Yes	186 (81.9)	90 (82.6)	96 (81.4)	
Ear fullness				0.676
No	76 (33.5)	38 (34.9)	38 (32.2)	
Yes	151 (66.5)	71 (65.1)	80 (67.8)	
Vertigo				0.407
No	202 (89.0)	95 (87.2)	107 (90.7)	
Yes	25 (11.0)	14 (12.8)	11 (9.3)	
Dizziness				0.243
No	161 (70.9)	73 (67.0)	88 (74.6)	
Yes	66 (29.1)	36 (33.0)	30 (25.4)	
Headache				
No	216 (95.2)	104 (95.4)	112 (94.9)	>0.999
Yes	11 (4.8)	5 (4.6)	6 (5.1)	
History of ear diseases				
No	214 (94.3)	102 (93.6)	112 (94.9)	0.778
Yes	13 (5.7)	7 (6.4)	6 (5.1)	
Brain MRI abnormalities				>0.999
No	213 (93.8)	102 (93.6)	111 (94.1)	
Yes	14 (6.2)	7 (6.4)	7 (5.9)	
Underlying disease				
Hypertension				0.667
No	204 (89.9)	99 (90.8)	105 (89.0)	
Yes	23 (10.1)	10 (9.2)	13 (11.0)	
Diabetes mellitus				0.762
No	216 (95.2)	103 (94.5)	113 (95.8)	
Yes	11 (4.8)	6 (5.5)	5 (4.2)	
Therapeutic method				
Glucocorticoid therapy				
No	5 (2.2)	3 (2.8)	2 (1.7)	0.673
Yes	222 (97.8)	106 (97.2)	116 (98.3)	
Neurotrophic therapy				
No	2 (0.9)	0 (0.0)	2 (1.7)	0.499
Yes	225 (99.1)	109 (100.0)	116 (98.3)	
Tinnitus specific medication				
No	140 (61.7)	76 (69.7)	64 (54.2)	0.020
Yes	87 (38.3)	33 (30.3)	54 (45.8)	
Psychotropic medication				
No	128 (56.4)	53 (48.6)	75 (63.6)	0.032
Yes	99 (43.6)	56 (51.4)	43 (36.4)	
Chinese patent medicine				0.047
No	118 (52.0)	49 (45.0)	69 (58.5)	
Yes	109 (48.0)	60 (55.0)	49 (41.5)	
Thrombolysis				0.008
No	117 (51.5)	46 (42.2)	71 (60.2)	
Yes	110 (48.5)	63 (57.8)	47 (39.8)	
Local injection administration				
Intratympanic injection therapy				0.075
No	143 (63.0)	62 (56.9)	81 (68.6)	
Yes	84 (37.0)	47 (43.1)	37 (31.4)	
Postauricular injection therapy				0.160
No	188 (82.8)	86 (78.9)	102 (86.4)	
Yes	39 (17.2)	23 (21.1)	16 (13.6)	
Traditional Chinese Medicine therapy				
Chinese herbal medicine				0.281
No	56 (24.7)	23 (21.1)	33 (28.0)	
Yes	171 (75.3)	86 (78.9)	85 (72.0)	
Auricular acupressure with seeds				0.585
No	213 (93.8)	101 (92.7)	112 (94.9)	
Yes	14 (6.2)	8 (7.3)	6 (5.1)	
Acupoint application therapy				0.573
No	73 (32.2)	33 (30.3)	40 (33.9)	
Yes	154 (67.8)	76 (69.7)	78 (66.1)	
Herbal Hot Compress				0.573
No	73 (32.2)	33 (30.3)	40 (33.9)	
Yes	154 (67.8)	76 (69.7)	78 (66.1)	
Thunder fire moxibustion				0.458
No	34 (15.0)	14 (12.8)	20 (16.9)	
Yes	193 (85.0)	95 (87.2)	98 (83.1)	
Acupuncture				0.230
No	167 (73.6)	76 (69.7)	91 (77.1)	
Yes	60 (26.4)	33 (30.3)	27 (22.9)	
Tinnitus sound therapy				0.226
No	209 (92.1)	103 (94.5)	106 (89.8)	
Yes	18 (7.9)	6 (5.5)	12 (10.2)	
Vestibular compensation training				0.349
No	207 (91.2)	97 (89.0)	110 (93.2)	
Yes	20 (8.8)	12 (11.0)	8 (6.8)	
Laboratory tests (peripheral blood)				
WBC (median[IQR]),×10^9^/L	7.91 [6.17, 10.14]	7.98 [6.38, 10.56]	7.82 [5.82, 9.91]	0.379
NEUT (median[IQR])%	72.80 [61.35, 82.70]	71.20 [61.00, 82.70]	73.90 [61.73, 82.68]	0.719
PLT (median[IQR]) × 10^9^/L	254.00 [222.50, 293.00]	254.00 [218.00, 302.00]	254.00 [224.25, 278.00]	0.486
MPV (median[IQR]), fL	9.50 [9.00, 10.20]	9.30 [8.80, 10.10]	9.55 [9.10, 10.40]	0.033
PT (median[IQR]), s	12.80 [12.20, 13.40]	12.80 [12.20, 13.50]	12.80 [12.12, 13.40]	0.621
APTT (median[IQR]), s	27.40 [25.55, 29.70]	26.90 [24.60, 29.50]	27.70 [26.22, 29.90]	0.035
TT (median[IQR]), s	15.30 [14.50, 16.10]	15.10 [14.40, 16.00]	15.40 [14.72, 16.10]	0.113
FIB (median[IQR]), g/L	2.56 [2.24, 2.94]	2.60 [2.32, 3.01]	2.53 [2.24, 2.81]	0.260
K (median[IQR]), mmol/L	4.10 [3.80, 4.30]	4.01 [3.80, 4.30]	4.10 [3.85, 4.30]	0.344
Na (median[IQR]), mmol/L	141.81 [140.00, 143.00]	142.00 [140.00, 143.00]	141.00 [140.00, 142.75]	0.473
Cl (median[IQR]), mmol/L	106.00 [104.00, 108.00]	106.00 [104.00, 108.00]	106.00 [104.00, 107.00]	0.669
Ca (median[IQR]),mmol/L	2.32 [2.26, 2.39]	2.32 [2.26, 2.38]	2.32 [2.26, 2.39]	0.951
GLU (median[IQR]), mmol/L	6.18 [5.36, 7.34]	6.21 [5.27, 7.09]	6.18 [5.41, 7.41]	0.359
BUN (median[IQR]), mmol/L	4.93 [4.20, 5.70]	5.26 [4.34, 6.17]	4.81 [4.06, 5.50]	0.013
Cr (median[IQR]), μmol/L	59.00 [49.00, 71.00]	57.00 [49.00, 68.60]	59.60 [49.25, 73.22]	0.421
TP (median[IQR]), g/L	70.70 [66.60, 74.15]	70.70 [65.70, 74.70]	70.80 [67.17, 73.75]	0.880
ALB (median[IQR]), g/L	44.60 [42.15, 46.40]	44.50 [41.80, 46.10]	44.65 [42.40, 46.77]	0.273
ALT (median[IQR]), U/L	17.30 [12.90, 29.25]	18.10 [13.70, 29.60]	16.65 [12.40, 28.72]	0.250
AST (median[IQR]), U/L	18.60 [15.25, 23.50]	18.90 [15.60, 22.40]	18.45 [14.83, 24.53]	0.799
ALT/AST (median[IQR])	0.95 [0.77, 1.41]	0.97 [0.81, 1.41]	0.92 [0.73, 1.42]	0.271

DD, Disease duration; LOS, Length of hospital stay; WBC, White Blood Cell; NEUT, Neutrophil; PLT, Platelet; MPV, Mean Platelet Volume; PT, Prothrombin Time; APTT, Activated Partial Thromboplastin Time; TT, Thrombin Time; FIB, Fibrinogen; K, Potassium; Na, Sodium; Cl, Chloride; Ca, Calcium; GLU, Glucose; BUN, Blood Urea Nitrogen; Cr, Creatinine; TP, Total Protein; ALB, Albumin; ALT, Alanine Aminotransferase; AST, Aspartate Aminotransferase; ALT/AST, Alanine Aminotransferase/Aspartate Aminotransferase Ratio.

### 3.3 Feature selection

Feature selection was conducted exclusively within the training set to prevent information leakage. All candidate predictors were first entered into the initial XGBoost model, and predictors were ranked according to XGBoost-derived importance scores. Multiple top-ranked feature subsets were then compared.

The final four-variable XGBoost model incorporating APTT, disease duration, PLT, and TP achieved the highest validation AUC among the evaluated feature subsets, providing an optimal balance between predictive performance and model parsimony.

To assess the stability of feature selection, a bootstrap-based variable stability analysis was performed using 100 resampled datasets. APTT and disease duration emerged as the most robust core predictors, with top-10 frequencies of 0.71 and 0.72, respectively. PLT also demonstrated high stability with a top-10 frequency of 0.58. In contrast, TP showed a relatively lower top-10 frequency of 0.48, indicating that while it contributes to the model, its importance is less stable across different data subsets compared to the other three features (Supplementary Table S3).

To further evaluate the incremental contribution of TP, a sensitivity analysis was performed. The validation AUC of a three-variable model (excluding TP) was 0.706 (95% CI: 0.576–0.837), which was marginally lower than the 0.718 (95% CI: 0.590–0.846) achieved by the four-variable model. These findings suggest that while APTT, disease duration, and PLT are the primary stable predictors, the inclusion of TP provides a slight improvement in predictive performance, justifying its retention in the final model for SHAP-based interpretation.

With a focused selection of 4 core variables against 109 event cases, our model maintains an EPV ratio of approximately 27. This significantly exceeds the conservative threshold of 10, effectively mitigating the risk of overfitting and ensuring the reproducibility of our prognostic signatures.

### 3.4 SHAP analysis and model interpretability

**Figure 3:**
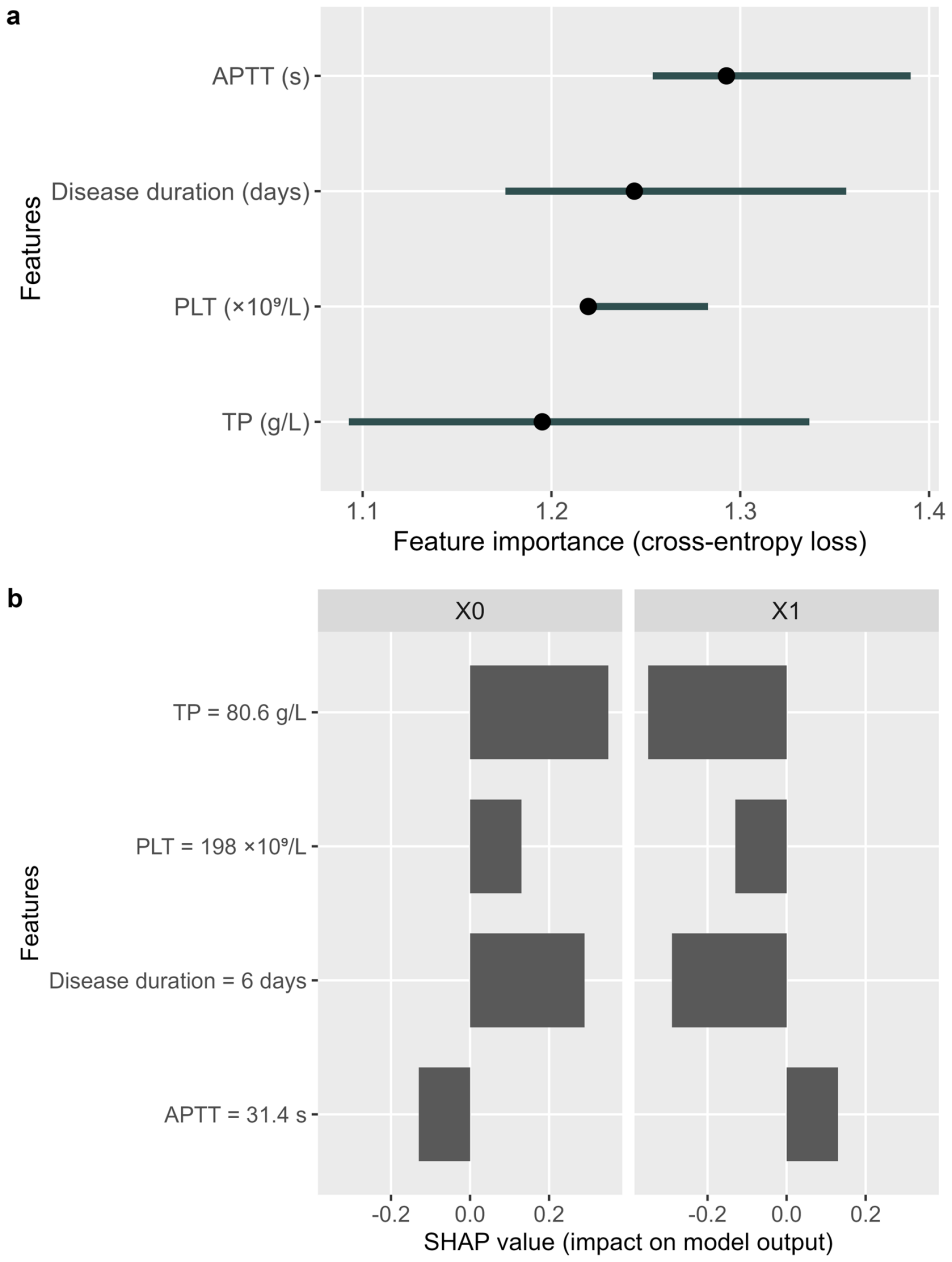
**(a)** Global feature importance ranking (by mean SHAP value); **(b)** Single-sample feature contribution (SHAP value). APTT, activated partial thromboplastin time; PLT, platelet (count); TP, total protein.

**Figure 4:**
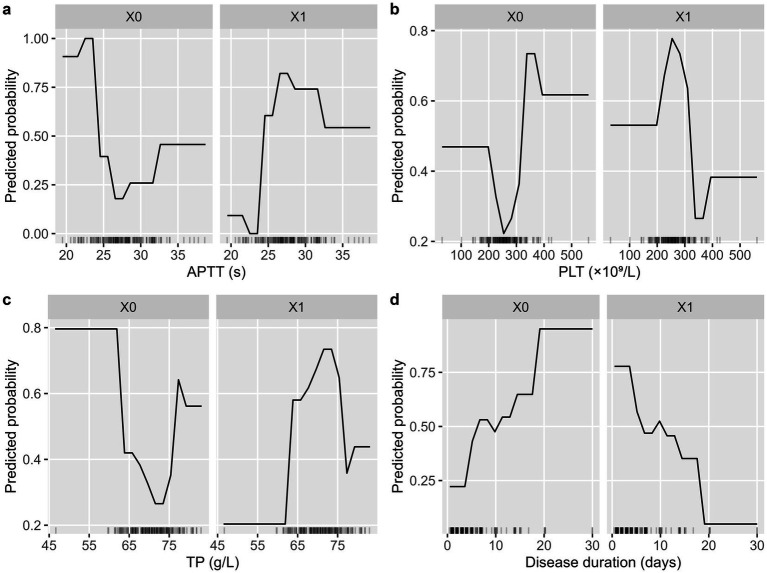
Single-feature impact curves of core factors on treatment outcomes. **(a)** APTT, activated partial thromboplastin time; **(b)** PLT, platelet (count); **(C)** TP, total protein; **(d)** Disease duration; X0 indicates treatment failure, and X1 indicates treatment response. The curves illustrate the association between each core feature and the predicted probability of treatment outcome.

**Figure 5:**
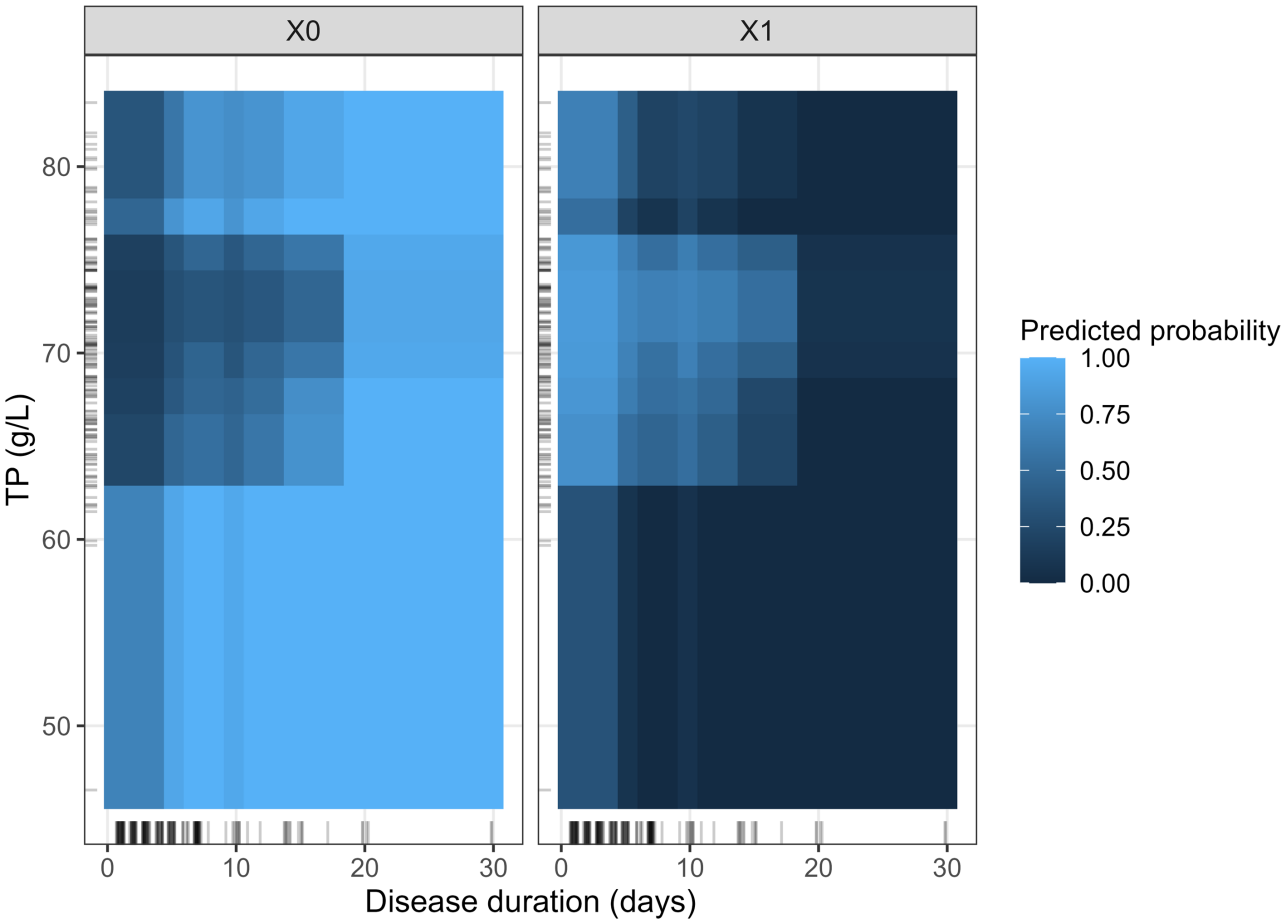
Dual-feature interaction heatmap of TP and disease duration. TP, total protein. X0 indicates treatment failure, and X1 indicates treatment response. The color intensity of the heatmap represents the predicted probability, with darker colors indicating higher probabilities.

SHAP analysis was performed to elucidate the contribution of individual features to model predictions (Figures 3–5).

#### 3.4.1 Global feature importance

The cumulative contribution of the four core features reached 65.8% (Figures 3a). Feature importance ranked as follows: APTT (1.46), disease duration (1.34), PLT (1.31), and TP (1.26).

#### 3.4.2 Single-feature marginal effects

SHAP dependence plots elucidated distinct non-linear relationships and defined specific “prognostic windows” for the four core parameters (Figure 4). Specifically, APTT exhibited an inverted U-shaped relationship with the predicted treatment outcome, characterized by an optimal window of 25–30 s and a precipitously increased risk of predicted treatment failure when values fell below 25 s. Next, a distinct threshold effect was identified for disease duration: the predicted risk of treatment failure remained stable within 10 days of symptom onset but rose sharply when clinical intervention was delayed beyond this 10-day threshold. Furthermore, PLT demonstrated a robust non-linear association with prognosis, with the predicted probability of a favorable response peaking within the 200–250 × 10^9^ /L range, whereas values exceeding 300 × 10^9^ /L were associated with a significantly elevated risk of treatment failure. Finally, TP displayed a significant non-linear relationship with treatment efficacy; predicted recovery probability was lowest below 60 g/L, peaked within the 65–75 g/L optimal prognostic window, and gradually declined at levels >75 g/L.

#### 3.4.3 Two-feature interaction effects

Interaction analysis revealed a joint effect between TP and disease duration (Figure 5). Favorable outcomes were more likely in patients with shorter disease duration and lower TP levels, whereas prolonged duration combined with elevated TP was associated with treatment failure.

#### 3.4.4 Single-sample contribution

The SHAP force plot of a representative case demonstrated feature contribution patterns consistent with global results (Figures 3b).

### 3.5 Predictive performance, calibration, and clinical utility of the models

**Figure 6:**
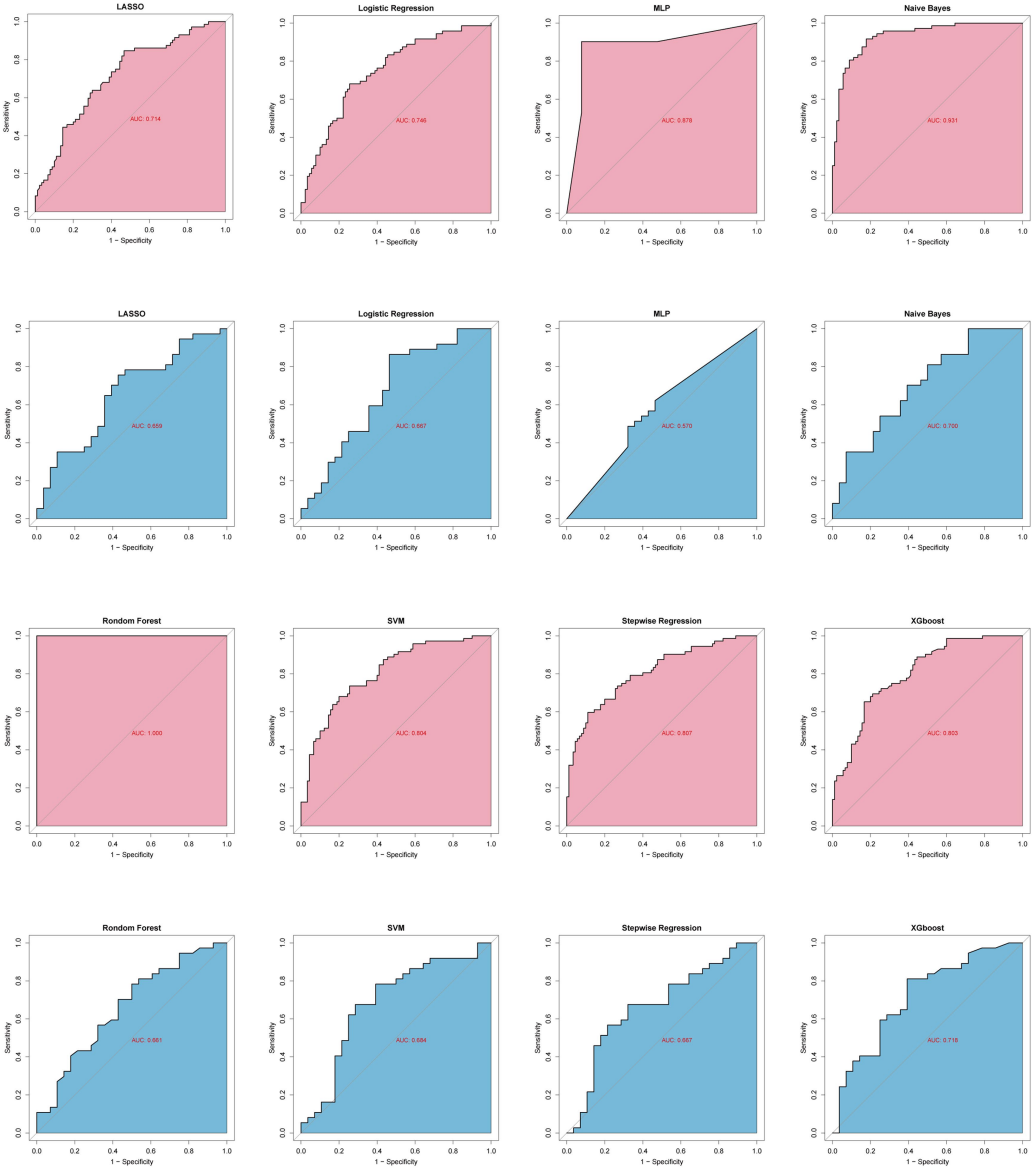
ROC curves of 8 machine learning models. Red curves represent the training set, and blue curves represent the validation set. The AUC values of each model in the two datasets are displayed in the graph. LASSO, Least Absolute Shrinkage and Selection Operator; XGBoost, Extreme Gradient Boosting; SVM, Support Vector Machine; MLP, Multi-Layer Perceptron; Naive Bayes, Naive Bayes Classifier.

ROC analysis (Figure 6; Table 2) showed that the NB model achieved the highest AUC in the training set (0.931, 95% CI: 0.893–0.968), followed by the MLP model (0.878, 95% CI: 0.818–0.939). XGBoost and SVM demonstrated comparable performance.

**Table 2:** Performance of 8 machine learning models.

Model	AUC (95%CI)	F1	Accuracy	Sensitivity	Specificity
LASSO (train)	0.714 (0.635–0.793)	0.714	0.648	0.789	0.472
LASSO (test)	0.659 (0.523–0.795)	0.559	0.538	0.679	0.432
Logistic Regression (train)	0.746 (0.671–0.822)	0.725	0.673	0.778	0.542
Logistic Regression (test)	0.667 (0.528–0.806)	0.595	0.538	0.786	0.351
Random Forest (train)	1.000 (1.000–1.000)	1.000	1.000	1.000	1.000
Random Forest (test)	0.661 (0.525–0.796)	0.63	0.585	0.821	0.405
Stepwise Regression (train)	0.807 (0.740–0.874)	0.77	0.735	0.8	0.653
Stepwise Regression (test)	0.667 (0.530–0.804)	0.625	0.631	0.714	0.568
XGBoost (train)	0.803 (0.737–0.869)	0.783	0.747	0.822	0.653
XGBoost (test)	0.718 (0.590–0.846)	0.6	0.569	0.75	0.432
SVM (train)	0.804 (0.737–0.871)	0.77	0.735	0.8	0.653
SVM (test)	0.684 (0.546–0.823)	0.62	0.585	0.786	0.432
MLP (train)	0.878 (0.818–0.939)	0.922	0.914	0.922	0.903
MLP (test)	0.570 (0.437–0.704)	0.567	0.554	0.679	0.459
Naive Bayes (train)	0.931 (0.893–0.968)	0.859	0.846	0.844	0.847
Naive Bayes (test)	0.700 (0.571–0.830)	0.594	0.6	0.679	0.541

LASSO, Least Absolute Shrinkage and Selection Operator; XGBoost, Extreme Gradient Boosting; SVM, Support Vector Machine; MLP, Multi-Layer Perceptron; Naive Bayes, Naive Bayes Classifier.

In the validation set, XGBoost achieved the highest AUC among the candidate models (0.718, 95% CI: 0.590–0.846). This result indicates moderate discriminative ability, although the relatively wide confidence interval reflects uncertainty related to the limited validation sample size. In contrast, RF and MLP exhibited substantial performance declines from the training set to the validation set, suggesting overfitting. In particular, the high training-set AUC of RF should be interpreted as apparent performance rather than evidence of true generalization ability (Supplementary Table S4).

**Figure 7:**
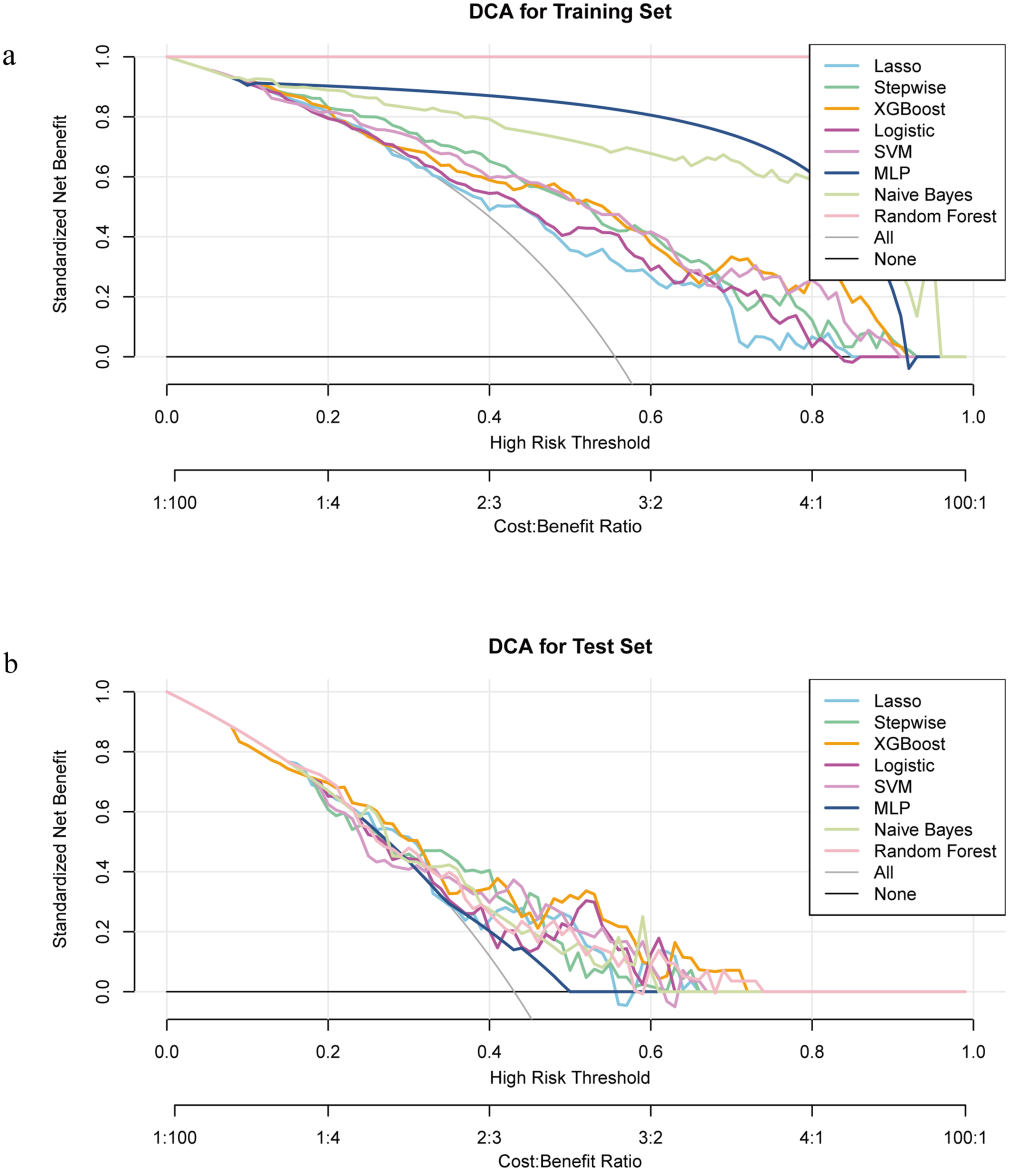
Decision curves of 8 machine learning models (a = training set, b = validation set). LASSO, Least Absolute Shrinkage and Selection Operator; XGBoost, Extreme Gradient Boosting; SVM, Support Vector Machine; MLP, Multi-Layer Perceptron; Naive Bayes, Naive Bayes Classifier.

DCA (Figure 7) suggested that the XGBoost model provided a relatively favorable net benefit compared with other models and baseline strategies across a range of threshold probabilities. Notably, while the MLP model showed high benefit in the training set, XGBoost exhibited more robust generalization in the validation set, confirming its clinical utility for individualized treatment decision-making in SSNHL patients.

**Figure 8:**
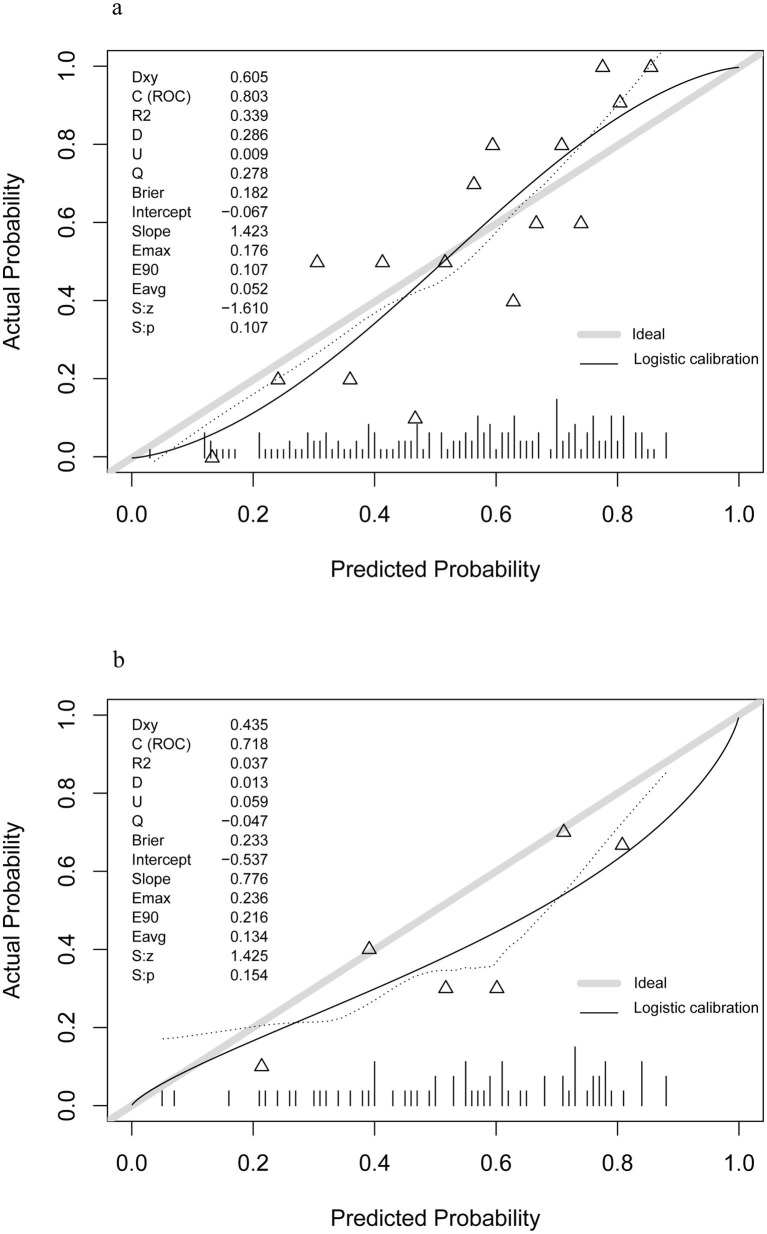
Calibration curves of the XGBoost model. **(a)** Training set; **(b)** validation set; C(ROC), C-statistic (Receiver Operating Characteristic Curve). Dxy, Discrimination Index; R2, R-squared; D, Discrimination slope; U, Unreliability; Q, Quality; Brier, Brier Score; Emax, Maximum absolute error; E90, 90th percentile of absolute errors; Eavg, Average absolute error; S:z, Spiegelhalter Z-statistic; S:p, Spiegelhalter *p*-value.

Calibration analysis (Figure 8) revealed acceptable agreement between predicted and observed outcomes. The Brier scores were 0.182 for the training set and 0.233 for the validation set. However, the validation cohort showed some deviation, particularly in the high-risk range (calibration slope = 0.776). Therefore, the calibration performance should be interpreted as preliminary and requires further assessment in larger external cohorts.

This study developed and validated multiple machine learning models to predict prognosis in SSNHL patients receiving integrated therapy.

## 4 Discussion

While the current XGBoost-based framework demonstrates acceptable discriminative performance (AUC 0.718), several limitations remain for its direct clinical application. Based on SHAP and variable stability analyses, APTT and disease duration were identified as the most robust core predictors, whereas PLT and TP provided important but supplementary prognostic value. The identification of these systemic indicators highlights the potential of using routine baseline clinical data for early risk stratification in SSNHL. Specifically, our bootstrap-based stability analysis confirmed that although all four variables contribute to the model, APTT and disease duration show the highest reliability across different data subsets, while TP serves as a supplementary marker that slightly enhances predictive performance.

Notably, our focus on the “Qi stagnation and blood stasis” phenotype represents a form of clinical stratification. This approach effectively reduces population heterogeneity and, together with a robust Events Per Variable (EPV) ratio of approximately 27, significantly lowers the risk of overfitting. Compared with previous studies based on single treatment modalities, the present study reflects real-world integrated TCM-WM treatment settings, thereby enhancing its clinical relevance. To ensure methodological rigor, strict dataset partitioning and validation set locking were applied, with all preprocessing and feature selection conducted exclusively within the training set to prevent information leakage. Nevertheless, these findings should be considered preliminary, and further multicenter validation is essential to translate this framework into a robust clinical decision-support tool.

In terms of model performance, several flexible models, particularly RF and MLP, showed marked discrepancies between training and validation performance, suggesting overfitting. The training-set AUCs reported in this study represent apparent performance because they were calculated by applying the fitted models back to the training data. Therefore, they may be optimistic and should not be interpreted as unbiased estimates of generalization ability. In contrast, XGBoost showed the best validation-set AUC among the candidate models and a relatively smaller performance gap. Nevertheless, its validation AUC indicated moderate rather than excellent discrimination, and the relatively wide confidence interval reflected uncertainty due to the limited validation sample size. Therefore, XGBoost was selected as the final exploratory model, but external validation is required before clinical implementation.

APTT was identified as the most influential predictor. SHAP analysis revealed a non-linear (inverted U-shaped) relationship between APTT and prognosis, suggesting an optimal range of 25–30 s. Values below 25 s were associated with an increased risk of poor outcomes. Previous studies have identified shortened APTT as a risk factor for unfavorable prognosis (16), and our findings further refine its clinically relevant range. Mechanistically, APTT reflects the intrinsic coagulation pathway and overall coagulation status (17, 18). A shortened APTT indicates a hypercoagulable state that may promote microthrombus formation in the terminal arteries of the inner ear, exacerbating ischemic injury, whereas excessive prolongation may increase the risk of microhemorrhage, potentially impairing recovery.

Disease duration emerged as a decisive temporal predictor in our model, with the 10-day mark identified as a pivotal prognostic inflection point. Our data demonstrate that the likelihood of therapeutic success remains relatively preserved within the first 10 days of symptom onset, but undergoes a precipitous decline once this 10-day threshold is surpassed. Previous studies have reported similar thresholds at 7 days or 2 weeks (9, 19), with discrepancies likely attributable to differences in study populations and treatment strategies. From a pathophysiological perspective, early-stage cochlear injury may be reversible, whereas prolonged ischemia leads to irreversible hair cell and neural damage (20), emphasizing the importance of early intervention.

Intriguingly, while PLT and TP showed no significant association in the initial univariable screen, the XGBoost architecture prioritized them as high-gain features for multivariate prognosis. This divergence indicates that XGBoost successfully captured intricate non-linear interactions and threshold-dependent biological relationships that are frequently masked in conventional linear regression, thereby enhancing the granularity of risk stratification in SSNHL.

Notably, analysis of Figure 4b identified PLT count as a core independent predictor with a robust non-linear association with treatment response. PDP analysis revealed that the probability of favorable recovery peaked when PLT ranged between 200–250 × 10^9^ /L, a trend further validated by our stratified univariable analysis: the intermediate PLT subgroup (200–300 × 10^9^ /L) achieved the highest treatment response rate (57.43%), whereas the high PLT subgroup (>300 × 10^9^ /L) exhibited the lowest efficacy (37.78%). This concordance between model-driven insights and clinical evidence underscores elevated PLT as a critical biomarker of systemic hypercoagulability (21). This association is likely mediated by complex interactions between platelet aggregation, blood viscosity, and microcirculatory alterations (22, 23). Mechanistically, this non-linear pattern reflects the dual role of platelets in inner ear homeostasis: while intermediate levels maintain vascular integrity and stable cochlear microperfusion, thrombocytosis likely exacerbates ischemic injury via microthrombus formation, and excessively low levels (<200 × 10^9^ /L) may compromise hemostatic stability and hinder tissue repair.

In the final four-variable model, TP showed a tri-phasic association with SSNHL recovery, with an apparent optimal prognostic window between 65–75 g/L. While the former often indicates a catabolic or malnourished state that impairs tissue repair and drug pharmacokinetics, the latter may reflect hemoconcentration or chronic inflammation, both of which exacerbate blood viscosity and microcirculatory compromise in the stria vascularis (24–26). Consequently, a TP level within the 65–75 g/L range likely reflects a state of metabolic and hemodynamic homeostasis conducive to optimizing cochlear perfusion, highlighting the potential value of integrating nutritional and rheological biomarkers into precision prognostic frameworks. However, the bootstrap-based stability analysis indicated that TP (top-10 frequency: 0.48) was less stable than APTT, disease duration, and PLT, and a three-variable sensitivity model excluding TP achieved a similar validation AUC. Therefore, the role of TP should be interpreted cautiously as a potentially supplementary predictor rather than a robust core predictor, and its independent prognostic value needs to be further validated in larger multicenter cohorts.

The integration of APTT, disease duration, PLT, and TP as core predictors provides a comprehensive biological footprint of the “Qi stagnation and blood stasis” syndrome, offering objective clinical evidence for the TCM axiom: “Normal flow of Qi ensures blood circulation, while stagnation leads to stasis.” Mechanistically, shortened APTT reflects impaired “Qi-promoting function,” indicating a pro-stasis hypercoagulable state; elevated PLT constitutes the “tangible” material of stasis, obstructing cochlear microvessels; and abnormal TP levels signify disrupted “Qi-fluid” metabolism, resulting in increased blood viscosity. This integrative approach maps the pathophysiological progression from functional Qi stagnation to tangible blood stasis, supporting risk-adapted stratification and informing the targeted application of Qi-circulating and stasis-resolving therapies.

In comparison with previous studies, the model achieved an AUC of 0.718 with a relatively small sample size (*n* = 227), outperforming some earlier models (27, 28), and demonstrating comparable performance to larger cohort studies (29, 30). This suggests that controlling population heterogeneity may improve model efficiency even with limited sample size.

From a clinical perspective, the predictors retained in the final model are routinely available at hospital admission, without requiring specialized examinations such as electrocochleography or inner ear MRI (31). However, important audiological predictors, such as initial hearing threshold, hearing-loss severity, and audiogram configuration, were not available as structured predictors in the current modeling dataset. This may limit the clinical comprehensiveness of the model. The model achieved a sensitivity of 76.3%, indicating its potential utility in early risk stratification and screening of patients at high risk of poor prognosis. Clinically, this model may be applied at admission to assist in identifying high-risk patients and guiding early management decisions.

It should be noted that although multiple treatment-related variables were included in the analysis, none were retained in the final model. This suggests that, under a relatively standardized treatment framework, baseline physiological and laboratory indicators may play a dominant role in short-term prognosis. Nevertheless, this does not diminish the importance of individualized treatment, and further studies are needed to explore potential interactions between treatment strategies and baseline risk.

Importantly, although the predictive performance of the model is moderate, it emphasizes interpretability and clinical feasibility. To our knowledge, this is one of the few studies to develop an explainable machine learning model specifically for SSNHL patients under an integrated treatment setting, providing both predictive capability and clinically interpretable insights.

## 5 Limitations and future perspectives

While this framework demonstrates acceptable discriminative performance, several limitations remain. First, the single-center retrospective design may introduce selection bias and constrain external generalizability. To mitigate this, we have initiated a multicenter prospective study to validate and recalibrate the model in a larger cohort.

Second, total protein (TP) exhibited moderate stability in bootstrap resampling. Thus, it should be viewed as a supplementary mechanistic marker rather than a primary predictor. Its independent prognostic weight will be further scrutinized in our subsequent validation.

Third, although baseline audiometric data were available, they were intentionally excluded to specifically evaluate the independent prognostic potency of hematological and coagulation indicators. As detailed in our methodology, this design prioritizes the exploration of systemic biological signatures associated with “Qi stagnation and blood stasis,” independent of initial auditory damage. Future iterations will integrate these metrics to further refine predictive precision.

Fourth, the model’s applicability to other TCM classifications requires further investigation. Finally, as this study relies on internal validation, future research will focus on multi-omics and longitudinal biomarkers to transition this preliminary tool into a robust clinical decision-support system.

## 6 Conclusion

This study developed an explainable XGBoost-based prognostic model for SSNHL, demonstrating the feasibility of integrating machine learning into TCM-informed clinical decision-making. Our findings underscore the critical role of coagulation dynamics (APTT) and temporal factors (disease duration) in auditory recovery, mapping these parameters to the TCM framework of “Qi stagnation and blood stasis”. While offering a preliminary framework for risk stratification and reinforcing the importance of early intervention, these results must be interpreted with caution given the single-center nature and lack of external validation. Further multicenter prospective studies are essential to recalibrate the model for diverse populations and to validate its clinical utility in precision care pathways.

## Data Availability

Requests to access these datasets should be directed to GC, chengangxf@sina.com.
