# Supplementary material for: An explainable machine learning model for prognosis prediction in sudden sensorineural hearing loss under integrated therapy
Source: Front Med (Lausanne). 2026 Jun 1;13:1845137. doi: 10.3389/fmed.2026.1845137 (PMC13265344; doi:10.3389/fmed.2026.1845137)
Supplement: Supplementary file 1 [file Table_1.docx]

**Supplementary Material**

Supplementary Table S1. Baseline characteristics of patients in the modeling and validation groups

| **Characteristics** | **Modeling group (n=162)** | **Validation group (n=65)** | ***p-value*** |
| --- | --- | --- | --- |
| **Patient characteristics** |  |  |  |
| Sex |  |  | 0.164 |
| Male | 69 (42.6%) | 35 (53.8%) |  |
| Female | 93 (57.4%) | 30 (46.2%) |  |
| Age（median[IQR]）,years | 43.41 (15.12) | 43.28 (13.80) | 0.950 |
| DD（median[IQR]）, days | 4.00 [2.00, 7.00] | 4.00 [2.00, 7.00] | 0.469 |
| LOS（median[IQR]）, days | 7.00 [6.00, 10.00] | 7.00 [6.00, 9.00] | 0.383 |
| **Affected side** |  |  | 0.900 |
| Left | 79 (48.8%) | 33 (50.8%) |  |
| Right | 83 (51.2%) | 32 (49.2%) |  |
| **Associated symptom** |  |  |  |
| Tinnitus |  |  | 0.927 |
| No | 30 (18.5%) | 11 (16.9%) |  |
| Yes | 132 (81.5%) | 54 (83.1%) |  |
| Ear fullness |  |  | 0.482 |
| No | 57 (35.2%) | 19 (29.2%) |  |
| Yes | 105 (64.8%) | 46 (70.8%) |  |
| Vertigo |  |  | 0.437 |
| No | 142 (87.7%) | 60 (92.3%) |  |
| Yes | 20 (12.3%) | 5 (7.7%) |  |
| Dizziness |  |  | 0.846 |
| No | 116 (71.6%) | 45 (69.2%) |  |
| Yes | 46 (28.4%) | 20 (30.8%) |  |
| Headache |  |  | 0.811 |
| No | 155 (95.7%) | 61 (93.8%) |  |
| Yes | 7 (4.3%) | 4 (6.2%) |  |
| **History of ear diseases** |  |  | 0.888 |
| No | 152 (93.8%) | 62 (95.4%) |  |
| Yes | 10 (6.2%) | 3 (4.6%) |  |
| **Brain MRI abnormalities** |  |  | 0.756 |
| No | 151 (93.2%) | 62 (95.4%) |  |
| Yes | 11 (6.8%) | 3 (4.6%) |  |
| **Underlying disease** |  |  |  |
| Hypertension |  |  | 0.310 |
| No | 143 (88.3%) | 61 (93.8%) |  |
| Yes | 19 (11.7%) | 4 (6.2%) |  |
| Diabetes mellitus |  |  | 0.657 |
| No | 153 (94.4%) | 63 (96.9%) |  |
| Yes | 9 (5.6%) | 2 (3.1%) |  |
| **Therapeutic method** |  |  |  |
| Glucocorticoid therapy |  |  | >0.999 |
| No | 4 (2.5%) | 1 (1.5%) |  |
| Yes | 158 (97.5%) | 64 (98.5%) |  |
| Neurotrophic therapy |  |  | 0.909 |
| No | 2 (1.2%) | 0 (0.0%) |  |
| Yes | 160 (98.8%) | 65 (100.0%) |  |
| Tinnitus specific medication |  |  | 0.303 |
| No | 96 (59.3%) | 44 (67.7%) |  |
| Yes | 66 (40.7%) | 21 (32.3%) |  |
| Psychotropic medication |  |  | 0.584 |
| No | 89 (54.9%) | 39 (60.0%) |  |
| Yes | 73 (45.1%) | 26 (40.0%) |  |
| Chinese patent medicine |  |  | 0.834 |
| No | 83 (51.2%) | 35 (53.8%) |  |
| Yes | 79 (48.8%) | 30 (46.2%) |  |
| Thrombolysis |  |  | 0.240 |
| No | 79 (48.8%) | 38 (58.5%) |  |
| Yes | 83 (51.2%) | 27 (41.5%) |  |
| **local injection administration** |  |  |  |
| Intratympanic injection therapy |  |  | 0.438 |
| No | 99 (61.1%) | 44 (67.7%) |  |
| Yes | 63 (38.9%) | 21 (32.3%) |  |
| Postauricular injection therapy |  |  | >0.999 |
| No | 134 (82.7%) | 54 (83.1%) |  |
| Yes | 28 (17.3%) | 11 (16.9%) |  |
| **Traditional Chinese Medicine therapy** |  |  |  |
| Chinese herbal medicine |  |  | 0.874 |
| No | 39 (24.1%) | 17 (26.2%) |  |
| Yes | 123 (75.9%) | 48 (73.8%) |  |
| Auricular acupressure with seeds |  |  |  |
| No | 153 (94.4%) | 60 (92.3%) | 0.764 |
| Yes | 9 (5.6%) | 5 (7.7%) |  |
| Acupoint application therapy |  |  |  |
| No | 50 (30.9%) | 23 (35.4%) | 0.616 |
| Yes | 112 (69.1%) | 42 (64.6%) |  |
| Herbal Hot Compress |  |  |  |
| No | 50 (30.9%) | 23 (35.4%) | 0.616 |
| Yes | 112 (69.1%) | 42 (64.6%) |  |
| Thunder fire moxibustion |  |  |  |
| No | 28 (17.3%) | 6 (9.2%) | 0.183 |
| Yes | 134 (82.7%) | 59 (90.8%) |  |
| Acupuncture |  |  |  |
| No | 122 (75.3%) | 45 (69.2%) | 0.440 |
| Yes | 40 (24.7%) | 20 (30.8%) |  |
| **Tinnitus sound therapy** |  |  |  |
| No | 149 (92.0%) | 60 (92.3%) | >0.999 |
| Yes | 13 (8.0%) | 5 (7.7%) |  |
| **Vestibular compensation training** |  |  |  |
| No | 146 (90.1%) | 61 (93.8%) | 0.525 |
| Yes | 16 (9.9%) | 4 (6.2%) |  |
| **Laboratory tests (peripheral blood)** |  |  |  |
| WBC（median[IQR]）,×10⁹/L | 7.96 [6.10, 10.04] | 7.85 [6.48, 10.89] | 0.648 |
| NEUT（median[IQR]）% | 72.90 [61.25, 81.40] | 72.80 [61.50, 84.70] | 0.355 |
| PLT（median[IQR]）×10⁹/L | 257.50 [225.50, 293.00] | 249.00 [208.00, 283.00] | 0.226 |
| MPV（median[IQR]）, fL | 9.50 [9.00, 10.20] | 9.40 [8.90, 10.40] | 0.727 |
| PT（median[IQR]）, s | 12.80 [12.20, 13.40] | 12.80 [12.40, 13.40] | 0.605 |
| APTT（median[IQR]）, s | 27.40 [25.60, 29.78] | 27.30 [25.40, 29.60] | 0.967 |
| TT（median[IQR]）, s | 15.30 [14.50, 15.97] | 15.30 [14.40, 16.10] | 0.579 |
| FIB（median[IQR]）, g/L | 2.57 [2.28, 2.96] | 2.52 [2.16, 2.88] | 0.213 |
| K（median[IQR]）, mmol/L | 4.10 [3.80, 4.30] | 4.07 [3.80, 4.25] | 0.634 |
| Na（median[IQR]）, mmol/L | 142.00 [140.00, 143.00] | 141.00 [140.00, 142.00] | 0.070 |
| Cl（median[IQR]）, mmol/L | 106.00 [104.00, 108.00] | 106.00 [104.00, 107.00] | 0.802 |
| Ca（median[IQR]）,mmol/L | 2.32 [2.27, 2.39] | 2.32 [2.25, 2.38] | 0.524 |
| GLU（median[IQR]）, mmol/L | 6.12 [5.28, 7.00] | 6.61 [5.45, 7.62] | 0.063 |
| BUN（median[IQR]）, mmol/L | 4.88 [4.12, 5.68] | 5.06 [4.50, 6.01] | 0.179 |
| Cr（median[IQR]）, μmol/L | 57.00 [49.00, 70.97] | 61.00 [51.00, 74.00] | 0.236 |
| TP（median[IQR]）, g/L | 70.70 [66.67, 74.18] | 71.20 [66.50, 73.90] | 0.975 |
| ALB（median[IQR]）, g/L | 44.45 [42.12, 46.40] | 44.80 [42.20, 46.70] | 0.732 |
| ALT（median[IQR]）, U/L | 17.00 [13.12, 28.15] | 19.00 [12.80, 33.70] | 0.578 |
| AST（median[IQR]）, U/L | 18.45 [15.30, 23.17] | 19.60 [14.80, 23.70] | 0.741 |
| ALT/AST（median[IQR]） | 0.94 [0.77, 1.41] | 1.03 [0.82, 1.44] | 0.397 |

DD: Disease duration; LOS: Length of hospital stay; WBC: White Blood Cell; NEUT: Neutrophil; PLT: Platelet; MPV: Mean Platelet Volume; PT: Prothrombin Time; APTT: Activated Partial Thromboplastin Time; TT: Thrombin Time; FIB: Fibrinogen; K: Potassium; Na: Sodium; Cl: Chloride; Ca: Calcium; GLU: Glucose; BUN: Blood Urea Nitrogen; Cr: Creatinine; TP: Total Protein; ALB: Albumin; ALT: Alanine Aminotransferase; AST: Aspartate Aminotransferase; ALT/AST: Alanine Aminotransferase/Aspartate Aminotransferase Ratio.

Supplementary Table S2. Distributions of key continuous variables before and after multiple imputation by chained equations (MICE)

| **Variable** | **Before Imputation** | | **After Imputation** | |
| --- | --- | --- | --- | --- |
|  | Mean ± SD | Median (IQR) | Mean ± SD | Median (IQR) |
| DD, days | 5.74 ± 5.03 | 4 (2-7) | 5.74 ± 5.03 | 4 (2-7) |
| APTT, s | 27.61 ± 3.40 | 27.35 (25.48-29.73) | 27.61 ± 3.40 | 27.35 (25.48-29.73) |
| PLT×10⁹/L | 258.61 ± 60.73 | 254 (223-293) | 258.95 ± 61.20 | 254 (222.75-293) |
| TP, g/L | 70.62 ± 5.38 | 70.70 (66.60-74.10) | 70.59 ± 5.39 | 70.70 (66.58-74.13) |

DD: Disease duration; APTT: Activated Partial Thromboplastin Time; PLT: Platelet; TP: Total Protein; SD: Standard Deviation; IQR: Interquartile Range.

Supplementary Table S3. XGBoost variable importance analysis via bootstrap sampling for predicting prognosis in SSNHL

| **Variable** | **Mean Importance (SD)** | **Mean rank** | **Top-5 Freq** | **Top-10 Freq** |
| --- | --- | --- | --- | --- |
| APTT | 59.95 (30.10) | 7.11 | 56% | 71% |
| Disease duration | 55.33 (28.24) | 7.74 | 46% | 72% |
| BUN | 57.04 (29.38) | 7.85 | 47% | 73% |
| GLU | 49.34 (23.36) | 8.17 | 33% | 68% |
| AST | 48.95 (24.29) | 8.38 | 38% | 70% |
| Age | 48.44 (24.45) | 8.58 | 36% | 64% |
| WBC | 45.85 (25.50) | 9.36 | 36% | 65% |
| PLT | 47.25 (26.34) | 9.54 | 41% | 58% |
| PT | 42.77 (21.21) | 9.82 | 28% | 63% |
| Ca | 40.43 (23.61) | 11.05 | 20% | 51% |
| TP | 38.76 (24.37) | 11.75 | 26% | 48% |
| FIB | 36.51 (26.05) | 12.97 | 22% | 39% |
| ALB | 28.02 (19.25) | 15.44 | 6% | 25% |
| NEUT | 27.67 (18.80) | 15.67 | 8% | 28% |
| TT | 26.86 (19.32) | 16.30 | 7% | 29% |
| ALT/AST | 24.74 (16.39) | 16.38 | 8% | 20% |
| ALT | 23.21 (15.12) | 16.69 | 3% | 14% |
| K | 24.40 (18.00) | 16.95 | 7% | 20% |
| Cr | 23.82 (13.39) | 17.00 | 5% | 23% |
| Cl | 23.86 (19.08) | 17.31 | 4% | 26% |
| MPV | 22.22 (13.72) | 17.51 | 4% | 17% |
| Psychotropic Medication | 19.79 (18.13) | 20.35 | 5% | 13% |
| Tinnitus-Specific Medication | 20.15 (20.93) | 20.81 | 9% | 21% |
| LOS | 16.18 (12.34) | 21.18 | 2% | 6% |
| Na | 12.87 (9.71) | 23.15 | 0% | 2% |
| Intratympanic Injection Therapy | 10.59 (12.68) | 26.20 | 1% | 5% |
| Affected side | 5.81 (9.58) | 29.03 | 1% | 1% |
| Thrombolysis | 8.56 (15.40) | 29.92 | 1% | 5% |
| Chinese Patent Medicine | 5.38 (8.17) | 31.51 | 0% | 1% |
| Gender | 2.89 (4.58) | 31.71 | 0% | 1% |
| Dizziness | 3.59 (5.83) | 31.89 | 0% | 0% |
| Postauricular Injection Therapy | 5.62 (7.86) | 32.02 | 0% | 0% |
| Ear Fullness | 2.43 (5.59) | 32.76 | 0% | 0% |
| Tinnitus | 1.32 (2.42) | 34.06 | 0% | 0% |
| Vertigo | 1.69 (4.12) | 34.50 | 0% | 0% |
| Acupuncture | 4.35 (6.53) | 35.05 | 0% | 0% |
| Hypertension | 1.76 (3.62) | 35.53 | 0% | 0% |
| Chinese Herbal Medicine | 3.01 (5.33) | 38.21 | 0% | 0% |
| Headache | 0.01 (0.07) | 38.35 | 0% | 0% |
| Brain MRI Abnormalities | 0.31 (0.92) | 39.67 | 0% | 0% |
| Diabetes Mellitus | 0.03 (0.20) | 39.85 | 0% | 0% |
| Vestibular Compensation Training | 2.01 (4.46) | 40.24 | 0% | 0% |
| Acupoint Application Therapy | 1.40 (4.13) | 40.90 | 0% | 0% |
| History of Ear Diseases | 0.05 (0.49) | 41.68 | 0% | 0% |
| Thunder Fire Moxibustion | 1.92 (7.02) | 42.25 | 0% | 1% |
| Glucocorticoid Therapy | 0.01 (0.10) | 42.75 | 0% | 0% |
| Tinnitus Sound Therapy | 0.63 (1.93) | 43.22 | 0% | 0% |
| Neurotrophic Therapy | 0.00 (0.00) | 43.83 | 0% | 0% |
| Auricular Acupressure with Seeds | 0.22 (0.87) | 45.99 | 0% | 0% |
| Herbal Hot Compress | 0.19 (0.74) | 46.82 | 0% | 0% |

The importance analysis was performed using 100 bootstrap iterations. Variables are ranked by their Mean Rank (lower values indicate higher predictive importance). Mean Importance (SD): The average Gain score (importance) and its standard deviation across bootstrap samples. Top-5/10 Frequency: The proportion of bootstrap iterations in which the variable was ranked within the top 5 or top 10 most important features. DD: Disease duration; LOS: Length of hospital stay; WBC: White Blood Cell; NEUT: Neutrophil; PLT: Platelet; MPV: Mean Platelet Volume; PT: Prothrombin Time; APTT: Activated Partial Thromboplastin Time; TT: Thrombin Time; FIB: Fibrinogen; K: Potassium; Na: Sodium; Cl: Chloride; Ca: Calcium; GLU: Glucose; BUN: Blood Urea Nitrogen; Cr: Creatinine; TP: Total Protein; ALB: Albumin; ALT: Alanine Aminotransferase; AST: Aspartate Aminotransferase; ALT/AST: Alanine Aminotransferase/Aspartate Aminotransferase Ratio.

Supplementary Table S4: Cross-validation out-of-fold AUC performance of different machine learning models

| **Model** | **CV OOF AUC** | **95% CI** |
| --- | --- | --- |
| XGBoost | 0.640 | 0.536–0.744 |
| Random Forest | 0.710 | 0.615–0.806 |
| MLP | 0.540 | 0.432–0.648 |

The CV OOF AUC was calculated via 5-fold cross-validation, where each fold’s model was trained on the training subset and evaluated on the held-out fold subset. AUC: Area Under the Receiver Operating Characteristic Curve; CV: Cross-Validation; OOF: Out-of-Fold; CI: Confidence Interval; XGBoost: Extreme Gradient Boosting. MLP: Multi-Layer Perceptron;
